# Identifying Information Needs for Hirschsprung Disease Through Caregiver Involvement via Social Media: A Prioritization Study and Literature Review

**DOI:** 10.2196/jmir.9701

**Published:** 2018-12-21

**Authors:** Kristy DM Wittmeier, Kendall Hobbs-Murison, Cindy Holland, Elizabeth Crawford, Hal Loewen, Melanie Morris, Suyin Lum Min, Ahmed Abou-Setta, Richard Keijzer

**Affiliations:** 1 Department of Pediatrics and Child Health Max Rady College of Medicine, Rady Faculty of Health Sciences University of Manitoba Winnipeg, MB Canada; 2 Children's Hospital Research Institute of Manitoba Winnipeg, MB Canada; 3 Department of Surgery Max Rady College of Medicine, Rady Faculty of Health Sciences University of Manitoba Winnipeg, MB Canada; 4 Swish Productions Ltd Winnipeg, MB Canada; 5 Neil John McLean Library University of Manitoba Winnipeg, MB Canada; 6 George and Fay Yee Centre for Healthcare Innovation Winnipeg, MB Canada

**Keywords:** Hirschsprung disease, caregivers, social media, research, surgeons, surveys and questionnaires

## Abstract

**Background:**

Patient and public involvement in health research is important to produce relevant and impactful results.

**Objective:**

This paper aimed to prioritize and summarize Hirschsprung disease (HD)–related information needs among caregivers of children with HD and pediatric surgeons through partnership with a parent-initiated social media campaign.

**Methods:**

We conducted a Web-based survey with the 2 stakeholder groups to identify information needs. The caregiver survey was conducted through a global Web-based community, and the surgeon survey was distributed to members of the Canadian Association of Paediatric Surgeons (CAPS). We conducted a literature review to identify evidence on the prioritized topics.

**Results:**

Our findings showed that 54.9% (89/162) of the individuals completed the caregiver survey and 23.8% (52/218 listed members) of the pediatric surgeons completed the survey distributed through CAPS. Only 20% (18/89) of the caregivers reported being very satisfied or satisfied with the current HD-related resources. A final prioritized list of information needs included bowel management, nutrition and growth, infection, perianal irritation, gastrointestinal pain, surgical diagnostics, and surgical complications. In total, 87 studies were included in the literature review, which included the following: 8 reviews, 2 randomized controlled trials, 74 cohort studies, and 3 practice guidelines. Two priority issues identified by caregivers had only a single study that met the inclusion criteria, whereas 1 topic had none.

**Conclusions:**

With caregiver and surgeon input, we identified 7 information priority areas related to HD. A review of the literature on the priorities found little evidence to support the development of high-quality guidelines. More research is necessary to meet the information needs related to HD as identified by stakeholders.

## Introduction

For a family affected by Hirschsprung’s disease (HD), the first few years can be a roller coaster of total normalcy and repeated hospitalizations. To learn more about this illness I created an online community for families and patients living with HD.

Parent of child with HD

There is an increasing focus on patient-centered or patient-oriented research to improve the relevance and impact of research. Programs and strategies such as INVOLVE [1], the Patient-Centered Outcomes Research Institute [2], and the Strategy for Patient-Oriented Research [3] are collectively functioning to promote and support patient and public involvement in health research [4]. Patient-centered or patient-oriented research emphasizes the involvement of patients and members of the public throughout all phases of research in a way that reflects the principles of inclusiveness, support, mutual respect, and cobuilding [3,5]. There is potential for tokenistic involvement if the public is merely informed of research without the ability to contribute to decision making within a project [4]. Instead, a combined method is recommended where patients or members of the public are involved as decision-making members of the research team, and broader consultation is conducted to help ensure representative input [4,6]. The highest level of involvement is patient- or public-led research.

We have previously conducted a parent-partnered study that examined the reach and responsiveness of a Web-based community and social media campaign developed to connect families affected by HD [7]. This initial work demonstrated that this community was highly responsive and the campaign helped to connect families across the globe. We then jointly developed this study to complete a needs assessment via social media to prioritize information needs related to HD. A secondary aim was to identify and summarize the best available evidence for each of the identified priorities.

## Methods

### Study Design

Our research team included a parent partner, 3 pediatric surgeons, a pediatric surgical nurse practitioner, a knowledge translation researcher, and a clinical nurse, all of whom have experience working with children with rare diseases. We obtained approval for the study from the University of Manitoba Health Research Ethics Board and the Health Sciences Centre Pediatric Research Impact Committee, and all participants provided informed consent.

The study involved 3 stages, which were as follows: surveying HD caregivers and pediatric surgeons to identify priority information needs regarding HD management; prioritization of the information needs, and a literature review to summarize the existing literature. Survey results are reported with guidance from the Checklist for Reporting Results of Internet E-Surveys using internet analytics as previously described [8].

### Needs Assessment

#### Caregiver Survey

This survey was developed to prioritize the information needs of caregivers and those living with HD. The survey asked caregivers to describe the problems most frequently encountered in caring for a child with HD and their satisfaction with HD-related resources that were available at that time (Multimedia Appendix 1). We also asked parents about the format they preferred to receive the study results. The survey was conducted using Fluid Surveys (Ontario, Canada), allowing for secure and anonymous data collection. We launched the survey in November 2013. It was posted for 1 month on the HD Facebook page along with information about the study. Reminders to complete the survey were frequently posted to promote participation by the site administrator. Facebook “likes,” reposts, and other sharing mechanisms (eg, to other sites such as reachhd.org [9] and HD-related group pages on Facebook) were also used to increase reach. We used an affiliated HD organization as the survey landing page (reachhd.org) and collected Google Analytics data to track survey metrics [8].

#### Pediatric Surgeon Survey

A survey was also administered to all members of the Canadian Association of Paediatric Surgeons (CAPS) using their Web-based survey tool. The survey was available for 1 month and reminder emails were sent to promote participation. We collected surgeon demographics (eg, number of years in practice and number of patients they manage with HD) and the top 5 HD-related medical issues they encounter. CAPS members were surveyed a second time 4 months later to determine the resources they used to guide the management of their patients with HD (Multimedia Appendix 2).

One team member (KHM) conducted a content analysis of the results from both caregiver and CAPS surveys. Common themes of information needs were identified and categorized as priority issues for each stakeholder group. A pediatric surgical nurse practitioner (CH) reviewed and verified the prioritized information needs from the caregiver survey, whereas 2 of the pediatric surgeon team members (RK and MM) verified those from the surgeon survey. These issues were then merged to create 1 list of the top 7 most common issues identified by caregivers and pediatric surgeons.

### Merging Priorities

A modified Delphi approach was then used to seek consensus among team members on the prioritized list. This process combined direct discussion and 2 rounds of anonymous survey of the research team members [10]. An a priori decision to give primary importance to the caregiver-identified priorities was adhered to. The top 7 most common information needs were then presented back to the Web-based caregiver community and surgeon stakeholder groups for validation.

### Literature Review

We then conducted a literature review to identify evidence to address the top 7 prioritized issues. A health science librarian in collaboration with an expert in review methodology (AAS) developed the search strategy. A second librarian conducted an independent peer review of the search strategy using the Peer Review of Electronic Search Strategies checklist [11]. The search was limited to systematic reviews or clinical practice guidelines published since 2000 and randomized controlled trials, clinical trials, cohorts, or case series from January 2010 to March 2015. Databases searched included Ovid Medline, Ovid EMBASE, CENTRAL, and EBSCOhost CINAHL. Two reviewers (KHM and CH) screened titles and abstracts independently for inclusion based on the predetermined inclusion criteria (Multimedia Appendix 3). For studies that were accepted by both reviewers, full texts were obtained and evaluated independently. Conflicts were resolved through consensus or after discussion with a third reviewer.

## Results

### Needs Assessment

#### Caregiver Survey

Of those who consented to participate, 54.9% (89/162) completed the caregiver survey. Moreover, 66% of the surveys were completed within the first week of posting the link. Short-segment HD was the most common diagnosis (36/89, 40%) reported, and most patients had received a diagnosis within the first month of life (78/89, 88%; Table 1).

When asked about satisfaction with current HD-related resources, only 20% (18/89) were satisfied or very satisfied with current resources, 42% (37/89) somewhat satisfied and (34/89) 38% not very or not satisfied.

#### Canadian Association of Paediatric Surgeons Survey

**Figure 1:**
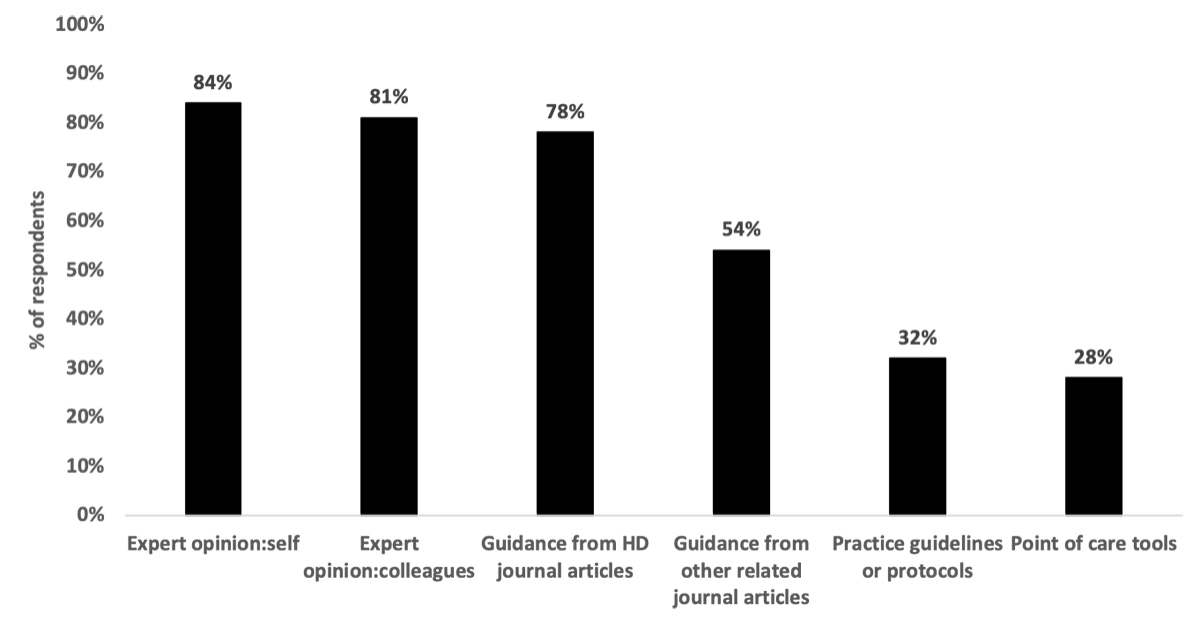
Pediatric surgeon's sources of information related to clinical Hirschsprung disease (HD) practice.

Of the 218 CAPS members, 23.8% (52/218) responded to the first survey and 46 (46/218, 21.1%) responded to the second survey (Table 1). The sources that Canadian pediatric surgeons report using to guide their clinical HD-related practice are shown in Figure 1.

### Merging Priorities

**Figure 2:**
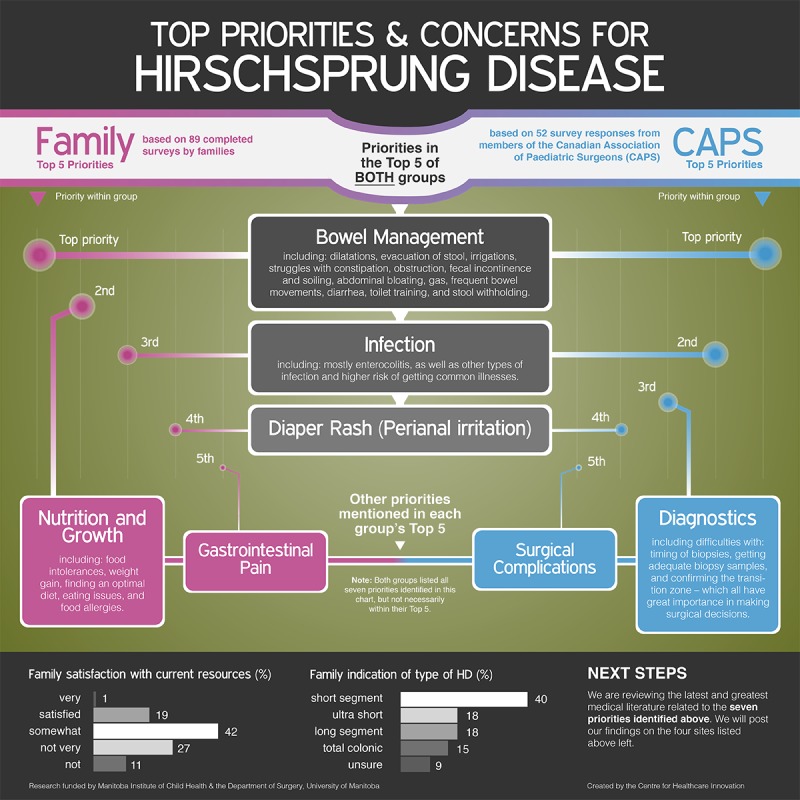
Infographic of top priorities and concerns related to Hirschsprung disease (HD).

The final top 7 priority information needs were summarized as an infographic (Figure 2) that was shared with the Web-based HD caregiver community through Facebook. Through a survey, community members were asked whether they agreed with the listed priority issues and for their feedback on the infographic format. Of the 96 individuals who provided feedback (90 caregivers, 6 people with HD), 91% (87/96) agreed or strongly agreed with the priority issues.

### Literature Review

We identified 8 reviews, 2 randomized trials, 59 retrospective cohort studies, 15 prospective cohort studies, and 3 practice guidelines that provided evidence on the 7 priority needs. The evidence is summarized by topic area in the following sections with emphasis on the highest levels of evidence in sections where a large number of studies were included (surgical complications and long-term outcomes).

### Bowel Management

One of the major problems faced by children with HD relates to bowel routines. Even after corrective surgery, many patients with HD still experience bowel management issues. In children who undergo surgery, systematic evaluation and the use of a structured and tailored approach to successfully treat persistent incontinence or soiling is recommended [12-14]. No studies covered the subtheme of toilet training specific to HD. Some authors suggest Botox injections to relax the sphincter muscle and promote defecation [15]. Single-center studies have also shown improvement in bowel function post-Soave with pelvic floor exercises [16] or Malone antegrade enemas [17]. All these interventions aim to achieve social continence.

### Infection

Hirschsprung’s associated enterocolitis (HAEC) is a serious, potentially life-threatening complication with an estimated incidence ranging from 4.6% to 54% [18]. Risk factors for developing HAEC are unclear. One systematic review that examined HAEC in relation to *Clostridium difficile* infection found 98 reported cases of HAEC related to *Clostridium difficile* infection from 1974 to 2014 [19]. There was insufficient data to analyze the role of other pathogens. One retrospective study found 58% of patients with HAEC had allergy to cow’s milk [20]. In terms of prevention, 1 report found no effect of probiotics administration when compared with placebo on HAEC incidence or recurrent HAEC postsurgery [21]. Similarly, a retrospective review found no difference in HAEC incidence or anastomotic stricture rates in children who either had or did not have routine anal dilations prescribed post the pull-through procedure [22].

With respect to surgical techniques, a systematic review reported a low incidence (10.2%) of postoperative HAEC after the transanal 1-stage pull-through procedure technique with HAEC successfully managed conservatively in majority of patients (81.5%) [18]. A single-center retrospective cohort study found that HAEC incidence decreased from 33.9% to 1.9% postsurgery with a transanal rectal mucosectomy and partial internal anal sphincterotomy [23]. We found no studies regarding caregivers’ concerns about the association between HD and susceptibility to common flus and colds.

### Perianal Irritation

One small (n=4) single-center pilot study suggested that the use of zinc oxide ointment with potato-derived protease inhibitors may reduce the otherwise intractable protease-induced perianal skin irritation in infants with long-segment HD [24].

### Nutrition and Growth

One single-center retrospective study found that growth and development in the first year of life were not different between infants with short-segment HD and those with long-segment HD [25].

### Gastrointestinal Pain

No studies that met our inclusion criteria were identified for this topic.

**Table 1:** Demographics of survey respondents.

Demographics					n (%)	
**HD^a^ community survey**						
	**Year of birth of individual living with HD (n=86)**					
			2012-2014			21 (24)
			2009-2011			34 (40)
			2006-2008			16 (19)
			1995-2005			13 (15)
			Before 1995			2 (2)
	**Type of HD (n=89)**					
				Ultrashort segment		16 (18)
				Short segment		36 (40)
				Long segment		16 (18)
				Total colonic		13 (15)
				Unsure		8 (9)
	**Age of HD diagnosis (n=89)**					
				0-1 months		78 (88)
				2-12 months		8 (9)
				13 months-4 years		2 (2)
				>4 years		1 (1)
**Pediatric surgeon survey^b^ (n=52)**						
	**Length of practice (years)**					
				0-5		11 (21)
				6-15		18 (35)
				>15		23 (44)
		**Cases of HD seen per year**				
				≤5		30 (58)
				>5		22 (42)
		**Multidisciplinary follow up offered in the clinic**				
				Yes		12 (23)

^a^HD: Hirschsprung disease.

^b^Surgeon demographics were similar for both surveys; demographics from the initial survey only are reported in Table 1.

### Diagnostics

#### Contrast Enema

Contrast enema (CE) has often been used as an adjunct to rectal biopsies for diagnosing HD. Barium or water-soluble contrast is instilled into the rectum to assess the transition zone. We found 10 articles published between 2006 and 2014 related to CE and its utility in HD [26-35]; five primary studies concluded that CE had either a low specificity, was not useful, or had a high false positive and negative rate [26,29-32]. A systematic review reported a sensitivity rate of 70% and specificity rate of 83% [28]. Wong et al [35] found that the addition of a delayed radiograph following CE raised the sensitivity from 69% to 100% but reduced the specificity from 89% to 78%. The literature suggests that CE is not as sensitive or specific as rectal biopsy to diagnose HD. The addition of a delayed film may increase sensitivity but lower specificity. CE may be helpful to raise suspicion of total colonic HD if certain criteria are found [35]. CE should not be used alone as a single method in the diagnosis of HD because it could be misleading and underestimate the extent of HD.

#### Rectal Biopsies

Classically, hematoxylin and eosin with or without acetylcholinesterase (AChE) are used in rectal biopsies to evaluate ganglion cells and nerve trunk hypertrophy respectively. Morris et al [36] state that calretinin immunohistochemistry may be superior to AChE in the context of total aganglionosis, superficial biopsies, and prematurity. Volpe et al [37] describe that with nerve hypertrophy, calretinin is a reliable marker for the transition zone. Uniformly, all authors concluded that calretinin immunohistochemistry is a reliable modality to diagnose HD and is equivalent if not superior to AChE with a sensitivity of 93.3% and specificity of 100% [38].

Several authors assessed different rectal biopsy techniques. Rectal suction biopsy (reserved for infants) [39], jumbo forceps [40], and full thickness biopsies [41] were all adequate to obtain tissue diagnosis for HD. Hematoxylin and eosin with or without AChE and with the addition of calretinin is effective in making the diagnosis via different techniques of procuring the rectal biopsy.

### Surgical Complications & Long-Term Outcomes

#### Complications

A 2013 systematic review [42] compared the outcomes between 444 transanal endorectal pull-through procedures and 348 conventional transabdominal approaches (including Soave, Duhamel, Swenson, and Rehbein procedures). Transanal endorectal pull-through procedures had shorter operative time and hospital stay, less postoperative soiling or incontinence and constipation, and no difference in postoperative enterocolitis. Similarly, Gosemann et al [43] found an advantage of the transanal over the open approaches in their systematic review.

Yang and Tang published in an abstract [44] the randomization of 54 children to a laparoscopic endorectal pull-through with a long or short cuff and reported that patients with a long cuff had a lower incidence of enterocolitis and better defecation in the first 6 months after surgery. The defecation frequency was similar 12 months after surgery. All authors considered a single stage approach standard of care for the treatment of short-segment HD. Most studies confirm the benefit of a transanal endorectal pull-through approach with or without laparoscopy over an open abdominal approach. Some studies reported that the transanal approach is associated with an increased rate of incontinence after the surgery [45,46]. Incidence rates of postoperative enterocolitis seem comparable among the different surgical approaches.

#### Long-Term Outcomes

Two prospective cohort studies evaluated the bowel function and quality of life in adults operated for HD during childhood. Ieiri et al [47] found that more than 85% of patients reported satisfactory bowel function (“good or excellent score”). Only 21.4% reported a normal score, and 16.7% and 19% reported incontinence and soiling, respectively. Jarvi et al performed a population-based study that included age- and sex-matched controls and reported that the overall bowel function score was lower in patients with HD, resulting in social problems associated with bowel function [48].

## Discussion

Research funders and public involvement organizations advocate for public participation to ensure that research is relevant, of high priority, and more easily translated into practice upon completion [1,5,49]. The public’s involvement can range from receiving the results of research findings to providing input and guidance to the research team to a partnership role as primary or coinvestigators [3,50,51]. In this study, a parent partnered with our research team, helping to set the agenda for the research and contributing to project decisions. The involvement of a parent on the research team and input from the survey respondents via a social media campaign were critical in highlighting areas in which more resources and research are needed to guide the diagnosis and care of people living with HD.

The James Lind Alliance is an organization that has developed standardized methods of involving the public in setting priorities for research around specific diagnoses [52]. Their priority-setting partnerships are designed to create a top 10 list of uncertainties in a given area to prioritize future research. In 2000, 1 study described a mismatch in funded research when compared with patient priorities [53]. More recently, Crowe et al [54] compared the James Lind Alliance recommended top 10 lists with registered trials and suggested that this mismatch has not yet been remedied. Registered research mainly focuses on drug treatments (37%-86%), whereas priority-setting partnerships mention drugs as a treatment priority less than 20% of the time [54]. We see a similar trend in this study. Nutrition and growth, perianal irritation, and gastrointestinal (GI) pain are the 2nd, 3rd, and 5th priority areas, respectively, identified by caregivers. Our literature search found only 1 study addressing nutrition and growth, 1 addressing perianal irritation, and no studies addressing GI pain in HD. Conversely, the topics that yielded the largest amount of information were diagnostics and surgical complications, which were the 3rd and 5th priorities of pediatric surgeons but not mentioned by caregivers. Thus, it is important for future research to consider both caregiver and health care provider priorities.

Our study demonstrates that it is feasible to engage with a global caregiver community online, via social media, to prioritize health research topics. We received responses from 89 caregivers; the majority responded within 1 week of posting the survey. Because HD is a rare disease, we would have been unable to recruit this large of a sample size from a single or even multicenter collaboration using more traditional methods within such a short period. However, it is prudent to acknowledge that the use of social media and an exclusively Web-based survey may have precluded the involvement of some individuals and therefore may not be entirely representative.

We undertook a number of measures to ensure that a rigorous literature review was conducted to identify studies addressing the prioritized areas, including a prespecified review methodology, peer-reviewed search strategy, and the inclusion of systematic review experts on our research team to oversee this process. A limitation is that we did not conduct a formal systematic review and as such may not have identified all of the relevant literature. Also, only English language studies were included. Furthermore, on the topic of surgical complications, owing to the large number of studies in this area, only specific study designs (systematic reviews, clinical practice guidelines, and randomized controlled trials) representing the highest level of evidence available were summarized in the evidence synthesis. Finally, within the individual topic areas, the reviewed research revealed limitations that include a large number of retrospective studies (often with small sample sizes) with a paucity of prospective research, data from large cohorts, or randomized trials. The use of nonstandardized outcome measures or clinical practice differences between sites further affected comparisons between studies.

In conclusion, involving caregivers through a parent-initiated social media campaign allowed us to identify and prioritize HD-related information needs of caregivers and surgeons. We have provided a summary of the evidence that is available to address these needs. Notably, the caregiver priority areas of nutrition and growth, perianal irritation, and GI pain have received very little attention in the literature to date. Future research should address these topics, in addition to the priority areas identified by surgeons, in collaboration with caregivers and individuals living with HD.

What began as a search for information resulted in establishing a support group. This has since grown into a hub for sharing the most recent medical information on HD. The HD community has partnered with the only HD not-for-profit organization: REACH (www.reachhd.org) and created a medical advisory board. This board consists of pediatric surgeons, researchers, geneticists, dieticians and gastroenterologists with a particular interest in HD. We use social media now not only to collect information but also to prioritize the information and share it so that families can have access to more than just emotional support.

Parent of a child with HD

## Appendix

Multimedia Appendix 1 Caregiver survey (prefaced with survey consent disclosure).

Multimedia Appendix 2 Pediatric surgeon survey (prefaced with survey consent disclosure).

Multimedia Appendix 3 Inclusion criteria.

## Abbreviations

AChE: acetylcholinesterase
CAPS: Canadian Association of Paediatric Surgeons
CE: contrast enema
GI: gastrointestinal
HAEC: Hirschsprung’s associated enterocolitis
HD: Hirschsprung disease

## References

[1] INVOLVE.

[2] (2016). Patient-Centred Oriented Research Institute (PCORI).

[3] (2014). Canadian Institutes of Health Research.

[4] Nass P, Levine S, Yancy C (2012). PCORI.

[5] (2014). PCORI.

[6] Oliver SR, Rees RW, Clarke-Jones L, Milne R, Oakley AR, Gabbay J, Stein K, Buchanan P, Gyte G (2008). A multidimensional conceptual framework for analysing public involvement in health services research. Health Expect.

[7] Wittmeier K, Holland C, Hobbs-Murison K, Crawford E, Beauchamp C, Milne B, Morris M, Keijzer R (2014). Analysis of a parent-initiated social media campaign for Hirschsprung's disease. J Med Internet Res.

[8] Eysenbach G (2004). Improving the quality of Web surveys: the Checklist for Reporting Results of Internet E-Surveys (CHERRIES). J Med Internet Res.

[9] REACH.

[10] Boulkedid R, Abdoul H, Loustau M, Sibony O, Alberti C (2011). Using and reporting the Delphi method for selecting healthcare quality indicators: a systematic review. PLoS One.

[11] McGowan J, Sampson M, Salzwedel D, Cogo E, Foerster V, Lefebvre C (2016). PRESS Peer Review of Electronic Search Strategies: 2015 Guideline Explanation and Elaboration.

[12] Levitt MA, Dickie B, Peña A (2012). The Hirschsprungs patient who is soiling after what was considered a “successful” pull-through. Semin Pediatr Surg.

[13] Chumpitazi BP, Nurko S (2011). Defecation disorders in children after surgery for Hirschsprung disease. J Pediatr Gastroenterol Nutr.

[14] Langer JC (2004). Persistent obstructive symptoms after surgery for Hirschsprung's disease: development of a diagnostic and therapeutic algorithm. J Pediatr Surg.

[15] Patrus B, Nasr A, Langer JC, Gerstle JT (2011). Intrasphincteric botulinum toxin decreases the rate of hospitalization for postoperative obstructive symptoms in children with Hirschsprung disease. J Pediatr Surg.

[16] Sun X, Wang R, Zhang L, Li D, Li Y (2012). Efficacy of pelvic floor muscle training for the treatment of fecal incontinence after Soave procedure for Hirschsprung disease. Eur J Pediatr Surg.

[17] Peeraully M, Lopes J, Wright A, Davies B, Stewart R, Singh S, More B (2014). Experience of the MACE procedure at a regional pediatric surgical unit: a 15-year retrospective review. Eur J Pediatr Surg.

[18] Ruttenstock E, Puri P (2010). Systematic review and meta-analysis of enterocolitis after one-stage transanal pull-through procedure for Hirschsprung's disease. Pediatr Surg Int.

[19] Mc Laughlin D, Friedmacher F, Puri P (2014). The impact of Clostridium difficile on paediatric surgical practice: a systematic review. Pediatr Surg Int.

[20] Umeda S, Kawahara H, Yoneda A, Tazuke Y, Tani G, Ishii T, Goda T, Hirano K, Ikeda K, Ida S, Nakayama M, Kubota A, Fukuzawa M (2013). Impact of cow's milk allergy on enterocolitis associated with Hirschsprung's disease. Pediatr Surg Int.

[21] El-Sawaf M, Siddiqui S, Mahmoud M, Drongowski R, Teitelbaum D (2013). Probiotic prophylaxis after pullthrough for Hirschsprung disease to reduce incidence of enterocolitis: a prospective, randomized, double-blind, placebo-controlled, multicenter trial. J Pediatr Surg.

[22] Aworanti O, Hung J, McDowell D, Martin I, Quinn F (2013). Are routine dilatations necessary post pull-through surgery for Hirschsprung disease?. Eur J Pediatr Surg.

[23] Zhang J, Li L, Hou W, Liu S, Diao M, Zhang J, Ming A, Cheng W (2014). Transanal rectal mucosectomy and partial internal anal sphincterectomy for Hirschsprung's disease. J Pediatr Surg.

[24] Berger S, Rufener J, Klimek P, Zachariou Z, Boillat C (2012). Effects of potato-derived protease inhibitors on perianal dermatitis after colon resection for long-segment Hirschsprung's disease. World J Pediatr.

[25] More K, Rao S, McMichael J, Minutillo C (2014). Growth and developmental outcomes of infants with hirschsprung disease presenting in the neonatal period: a retrospective study. J Pediatr.

[26] Chen JZ, Jamieson DH, Skarsgard ED (2010). Does pre-biopsy contrast enema delay the diagnosis of long segment Hirschsprung's disease?. Eur J Pediatr Surg.

[27] Das K, Kini U, Babu MK, Mohanty S, D'Cruz AJ (2010). The distal level of normally innervated bowel in long segment colonic Hirschsprung's disease. Pediatr Surg Int.

[28] de Lorijn F, Kremer L, Reitsma J, Benninga M (2006). Diagnostic tests in Hirschsprung disease: a systematic review. J Pediatr Gastroenterol Nutr.

[29] Flanagan S, Dietz C, Hoggard E (2011). Evaluation of the role of fluoroscopic enema diagnosis of Hirschsprung disease, clinical considerations and determining need for radiographic evaluation. Pediatric Radiology.

[30] Maerzheuser S, Bassir C, Rothe K (2012). Hirschsprung disease in the older child: diagnostic strategies. Clin Pediatr (Phila).

[31] Muller CO, Mignot C, Belarbi N, Berrebi D, Bonnard A (2012). Does the radiographic transition zone correlate with the level of aganglionosis on the specimen in Hirschsprung's disease?. Pediatr Surg Int.

[32] Saxena AM, Sodhi K, Rao K, Vaiphei K, Khandelwal N (2013). Role of contrast enema study in diagnosis of Hirschsprung's disease. Pediatric Radiology.

[33] Sheng T, Wang C, Lo W, Lien R, Lai J, Chang P (2012). Total colonic aganglionosis: Reappraisal of contrast enema study. Chinese Journal of Radiology (Taiwan).

[34] Wang C, Sheng T, Lo W, Lai J (2011). The difficulty in radiographic diagnosis of total colonic aganglionosis. Pediatric Radiology (Suppl).

[35] Wong A, Tsang D, Lam W (2014). How Useful is Contrast Enema in the Diagnosis of Hirschsprung’s Disease? Five-year Experience from a Local Referral Centre. Hong Kong J Radiol.

[36] Morris MI, Soglio DB, Ouimet A, Aspirot A, Patey N (2013). A study of calretinin in Hirschsprung pathology, particularly in total colonic aganglionosis. J Pediatr Surg.

[37] Volpe A, Alaggio R, Midrio P, Iaria L, Gamba P (2013). Calretinin, β-tubulin immunohistochemistry, and submucosal nerve trunks morphology in Hirschsprung disease: possible applications in clinical practice. J Pediatr Gastroenterol Nutr.

[38] Hiradfar M, Sharifi N, Khajedaluee M, Zabolinejad N, Taraz Jamshidi Shirin (2012). Calretinin Immunohistochemistery: An Aid in the Diagnosis of Hirschsprung's Disease. Iran J Basic Med Sci.

[39] Hayes CE, Kawatu D, Mangray S, LeLeiko NS (2012). Rectal suction biopsy to exclude the diagnosis of Hirschsprung disease. J Pediatr Gastroenterol Nutr.

[40] Hirsch BZ, Angelides AG, Goode SP, Garb JL (2011). Rectal biopsies obtained with jumbo biopsy forceps in the evaluation of Hirschsprung disease. J Pediatr Gastroenterol Nutr.

[41] Vollmer DD, Fair K, Hong YA, Beaudoin CE, Pulczinski J, Ory MG (2015). Apps seeking theories: results of a study on the use of health behavior change theories in cancer survivorship mobile apps. JMIR Mhealth Uhealth.

[42] Chen Y, Nah S, Laksmi N, Ong C, Chua J, Jacobsen A, Low Y (2013). Transanal endorectal pull-through versus transabdominal approach for Hirschsprung's disease: a systematic review and meta-analysis. J Pediatr Surg.

[43] Gosemann J, Friedmacher F, Ure B, Lacher M (2013). Open versus transanal pull-through for Hirschsprung disease: a systematic review of long-term outcome. Eur J Pediatr Surg.

[44] Yang L, Tang S (2013). A prospective study of laparoscopic transanal endorectal pull-through for subtotal colectomy in Hirschsprung's disease: Anastomosis using long cuff or short cuff?. Journal of Laparoendoscopic & Advanced Surgical Techniques.

[45] Romero P, Kroiss M, Chmelnik M, Königs I, Wessel LM, Holland-Cunz S (2011). Outcome of transanal endorectal vs. transabdominal pull-through in patients with Hirschsprung's disease. Langenbecks Arch Surg.

[46] Granström AL, Husberg B, Nordenskjöld A, Svensson P, Wester T (2013). Laparoscopic-assisted pull-through for Hirschsprung's disease, a prospective repeated evaluation of functional outcome. J Pediatr Surg.

[47] Ieiri S, Nakatsuji T, Akiyoshi J, Higashi M, Hashizume M, Suita S, Taguchi T (2010). Long-term outcomes and the quality of life of Hirschsprung disease in adolescents who have reached 18 years or older--a 47-year single-institute experience. J Pediatr Surg.

[48] Jarvi K, Laitakari E, Koivusalo A, Rintala R, Pakarinen M (2010). Bowel function and gastrointestinal quality of life among adults operated for Hirschsprung disease during childhood: a population-based study. Ann Surg.

[49] CIHR.

[50] Arnstein S (1969). A Ladder Of Citizen Participation. Journal of the American Institute of Planners.

[51] Tritter JQ, McCallum A (2006). The snakes and ladders of user involvement: Moving beyond Arnstein. Health Policy.

[52] (2016). James Lind Alliance.

[53] Tallon D, Chard J, Dieppe P (2000). Relation between agendas of the research community and the research consumer. Lancet.

[54] Crowe S, Fenton M, Hall M, Cowan K, Chalmers I (2015). Patients’, clinicians’ and the research communities’ priorities for treatment research: there is an important mismatch. Res Involv Engagem.

