# Supplementary material for: Identifying Information Needs for Hirschsprung Disease Through Caregiver Involvement via Social Media: A Prioritization Study and Literature Review
Source: J Med Internet Res. 2018 Dec 21;20(12):e297. doi: 10.2196/jmir.9701 (PMC6320415; doi:10.2196/jmir.9701)
Supplement: Multimedia Appendix 1 [file jmir_v20i12e297_app1.pdf]

Multimedia Appendix 1. Caregiver Survey (Prefaced with Survey Consent Disclosure)

**Part 1 – Demographics**	
What range does the year of birth of your child fall in?	2012-2014<br>2009-2011<br>2006-2008<br>1995-2005<br>Before 1995
What type of Hirschsprung's Disease (HD) does your child have?	Ultra Short Disease<br>Short Segment Disease (Includes the rectum up to the sigmoid colon)<br>Long Segment Disease (beyond the sigmoid colon)<br>Unsure
At what age was your child diagnosed with Hirschsprung's Disease?	0-1 month<br>2-12 months<br>13 months- 4 years<br>> 4 years
What age did your child have their "pull through" surgery?	0-3 months<br>>3months- 6 months<br>>6 months- 12 months<br>>12 months
Give up to 5 problems that your child has, or has had in the past, related to HD:	[open ended]<br>1.<br>2.<br>3.<br>4.<br>5.
What is your confidence level to successfully manage this problem?	Not Confident at all<br>Not Very Confident<br>Somewhat Confident<br>Confident<br>Very Confident
*Repeat as above for Problems #2- #5	
How satisfied are you with the current resources provided to you to manage problems that arise with your child's Hirschsprung's Disease? (web-based resources or written material available)	Not Satisfied<br>Not Very Satisfied<br>Somewhat Satisfied<br>Satisfied<br>Very Satisfied
	Comments:
If information was available on how to manage problems related to HD how would you like to obtain this information?	Please rank from 1- 5 (1 is the most desired and 5 is the least desired route)<br><br>__ Link to a health care site where written guidelines are available (i.e. Winnipeg Children's Hospital home page)<br>__ A report from this project (available on the HD social media sites)<br>__ In a story where a child and parent encounter and resolve a problem (available on the HD social media sites)<br>__ In a video explanation (I.e. Youtube)<br>__ Other: ________________________
