# Supplementary material for: Identifying Information Needs for Hirschsprung Disease Through Caregiver Involvement via Social Media: A Prioritization Study and Literature Review
Source: J Med Internet Res. 2018 Dec 21;20(12):e297. doi: 10.2196/jmir.9701 (PMC6320415; doi:10.2196/jmir.9701)
Supplement: Multimedia Appendix 2 [file jmir_v20i12e297_app2.pdf]

Multimedia Appendix 2. Pediatric Surgeon Survey (Prefaced with Survey Consent Disclosure)

**Part 1 – Survey 1**	
What are the most common problems (top 5) related to Hirschsprung's Disease that you encounter in your practice?	[open ended]<br>1.<br>2.<br>3.<br>4.<br>5.
How long have you been in practice with patients who have Hirschsprung's Disease?	0-5 years<br>6-15 years<br>>15 years
How many patients with Hirschsprung's Disease do you see every year?	0-5 cases<br>>5 cases
Do you have multidisciplinary follow up for Hirschsprung's Disease patients in your practice?	Yes<br>No
**Part 2 – Survey 2**	
What resources do you use to guide your medical management of HD?<br>*Check all that apply.*	Point of Care Tools (i.e. UpToDate, MD-consult)<br><br>Practice Guidelines or Protocol<br><br>Your Expert Opinion (knowledge & experience)<br><br>Expert Opinion from a Colleague<br><br>Journal Articles in fields related to HD:<br>o Systematic reviews / meta-analyses<br>o Narrative reviews<br>o Clinical trials<br>o Cohort studies<br>o Case studies<br>o Editorial / opinion papers<br>o Any study type<br><br>Journal Articles about HD:<br>o Systematic reviews / meta-analyses<br>o Narrative reviews<br>o Clinical trials<br>o Cohort studies<br>o Case studies<br>o Editorial / opinion papers<br>o Any study type<br><br>Other:
Do you have practice guidelines, tools or pathways that your facility uses for managing HD that you could share with us?	Yes<br>No<br>**If yes**, please send via email to our study team at [email provided].
May we contact you for further information on guidelines, tools, or pathways?	Yes<br>No<br>**If so,** please provide your preferred contact information.
	Contact information:
