# Supplementary material for: Identifying Information Needs for Hirschsprung Disease Through Caregiver Involvement via Social Media: A Prioritization Study and Literature Review
Source: J Med Internet Res. 2018 Dec 21;20(12):e297. doi: 10.2196/jmir.9701 (PMC6320415; doi:10.2196/jmir.9701)
Supplement: Multimedia Appendix 3 [file jmir_v20i12e297_app3.pdf]

Multimedia Appendix 3. Inclusion Criteria

- Relevant to HD.
- Post-corrective surgery, with exception to the theme of surgical diagnostics.
- Fits within a prioritized theme and address’ one of the developed key clinical questions
- Studies with specific sub-populations that have data separated were included while those that were not separated out in the data were only included if >50% of their population had HD.
- Abstract only without full publications were included.
- Under theme “Surgical Complications and Long Term Outcomes” studies were excluded if they used adolescent or adult operative techniques.
- Under theme “Surgical Complications and Long Term Outcomes” only the highest level of evidence was used in the summary.
